# METTL3-Mediated ADAMTS9 Suppression Facilitates Angiogenesis and Carcinogenesis in Gastric Cancer

**DOI:** 10.3389/fonc.2022.861807

**Published:** 2022-04-28

**Authors:** Nuofan Wang, Xinying Huo, Baoguo Zhang, Xiaoxiang Chen, Shuli Zhao, Xuesong Shi, Hao Xu, Xiaowei Wei

**Affiliations:** ^1^Department of General Surgery, The First Affiliated Hospital of Nanjing Medical University, Nanjing, China; ^2^Department of Oncology, Nanjing First Hospital, Nanjing Medical University, Nanjing, China; ^3^General Clinical Research Center, Nanjing First Hospital, Nanjing Medical University, Nanjing, China; ^4^Department of General Surgery, Nanjing First Hospital, Nanjing Medical University, Nanjing, China

**Keywords:** gastric cancer, N6-methyladenosine, METTL3, YTHDF2, ADAMTS9

## Abstract

The role of methyltransferase-like 3 (METTL3), which participates in catalyzing N-methyladenosine (m6A) RNA modification, in gastric cancer (GC) is unclear. Here, we found that METTL3 was overexpressed in human GC. Functionally, we verified that METTL3 promoted tumor cell proliferation and angiogenesis through a series of phenotypic experiments. Subsequently, ADAMTS9 was identified as the downstream effector of METTL3 in GC, which could be degraded by the YTHDF2-dependent pathway. Finally, the data suggested that METTL3 might facilitate GC progression through the ADAMTS9-mediated PI3K/AKT pathway. Our study unveiled the fundamental mechanisms of METTL3 in GC progression. The clinical value of METTL3 in GC deserves further exploration.

## Introduction

GC is the fifth most prevalent gastrointestinal malignancy globally (1). Although advancements in the treatment of GC have been achieved in recent years (2, 3), the prognosis of GC patients is still suboptimal. To date, the molecular pathogenesis of GC has not been well illustrated. It is essential to explore any critical cancer-related event that may contribute to the development of novel effective targeted therapies.

Increasing studies have shown that epigenetic modification strikingly contributes to GC progression, while major studies are focused on the transcriptional level (4–6). An increasing amount of evidence has demonstrated that m6A is the prevalent posttranscriptional modification in RNAs (7). Similar to other epigenetic processes, m6A modification is regulated by specific enzymes, including writers (methyltransferases), erasers (demethylases), and readers (m6A-binding proteins) (8). Aberrant expression of m6A regulators has been extensively identified in numerous diseases, including malignancies. For example, the core components of m6A writers, e.g., METTL14 and WTAP, can promote the progression of pancreatic and liver cancers (9–11). Erasers, including ALKBH5, have been reported to be tumor-suppressor genes (12, 13). Similarly, m6A reader proteins, such as YTHDF1/2/3, also participate in complex pathophysiological processes by identifying and binding to m6A motifs (14–20).

As a core component of the m6A writer, METTL3 is vital for m6A modification and has been identified as an oncogene in some hematological diseases and solid tumors (21–25). In myeloid leukemia, the inhibition of METTL3 has been proven to be a great potential medical strategy in the future. In the present study, we elucidated the promoting role of METTL3 in cell proliferation and angiogenesis, which are considered important in GC.

## Methods and Materials

### Clinical Samples and Public Data Collection

A total of 20 specimens, including tumor and adjacent normal tissues (ANTs), were obtained from GC patients receiving gastrectomy at Nanjing First Hospital from July 2019 to July 2020. No patient received antitumor treatment before surgery. The project was approved by the ethics committee of Nanjing First Hospital.

The public transcriptome data of METTL3 in GC were downloaded from The Cancer Genome Atlas (TCGA) database. The prognostic value of METTL3 in GC was evaluated in the Kaplan–Meier plotter database (http://kmplot.com/analysis/).

### Cell Transfection

Specific small hairpin RNAs (shRNAs) targeting METTL3 (shMETTL3#1, shMETTL3#2) or YTHDF2 (shYTHDF2) and the control group (shNC) were synthesized (GenePharma, Shanghai, China). pcDNA3.1 (Invitrogen, Carlsbad, CA, USA) was used to construct pcDNA-METTL3 and pcDNA-ADAMTS9. Lipofectamine 2000 (Invitrogen, USA) was employed to transfect cells following the manufacturer’s instructions. The sequence of shRNAs is listed in **Supplementary File 1**.

### Construction of Stable Knockdown and Overexpression Cells

Stable METTL3 knockdown and overexpression models were established by using a lentivirus vector system. Briefly, plasmid-cloned RelB cDNA and siRNA for knocking down METTL3 were transfected into AGC and HCG-27 cell lines according to the manufacturer’s instructions.

### Animal Experiments

For the formation of xenograft tumors, 5 × 10^6^ AGS cells mixed in Matrigel (BD Biosciences, Franklin Lakes, NJ, USA) were subcutaneously injected into BALB/c nude mice (5-week-old male). Tumor volumes were routinely measured every 5 days according to the following equation: V = 0.5 × (length × width^2^). One month later, the xenografts were surgically dissected after sacrificing the mice.

### Chick Chorioallantoic Membrane Assay

The chorioallantoic membrane (CAM) assay was employed to estimate the potential role of METTL3 in angiogenesis. The eggs were incubated in a humidified atmosphere of 80% at 38°C for 1 week. On embryonic day 8, 100 µl of a suspension of 1 × 10^6^ AGS cells in culture medium mixed with Matrigel was deposited on the surface of the CAM. The images were then taken, and the vessel areas were calculated.

### RNA-seq and meRIP-seq

For RNA-seq, RNA-seq of stable METTL3 knockdown AGS cells and their controls (NC) were performed with HiSeq-2500. The PolyA+ messenger RNA (mRNA) library was estimated with RNA-seq (H/M/R) Library Prep Kit. KEGG analysis was detected with DAVID.

For meRIP-seq, RNA fragmentation was performed by using NEBNext Magnesium RNA Fragmentation Module (NEB, Cambridge, UK). About 1/10 of the fragmented RNA was used as input control for RNA-seq by Genesky (Shanghai, China). The others were incubated with m6A antibody, and the enrichment of m6A containing mRNA was analyzed using high-throughput sequencing by Genesky (Shanghai, China).

### Statistical Analysis

All the data analyses were performed using SPSS 22.0 (IBM, Armonk, NY, USA). Our data are described as the mean ± S.D. Continuous variables with a normal distribution were analyzed using Student’s t-test. For multiple comparisons, the significant difference test was applied following ANOVA. The correlations were analyzed using Pearson’s correlation coefficients. A P value < 0.05 indicated statistical significance.

Cell culture, qRT-PCR, cell proliferation assays, tube formation assays, RNA stability, Western blots, RNA immunoprecipitation (RIP), m6A quantification, RNA-seq, and MeRIP-Seq are listed in **Supplementary File 1**.

## Results

### METTL3 Is Overexpressed and Related to Poor Prognosis in GC

**Figure 1:**
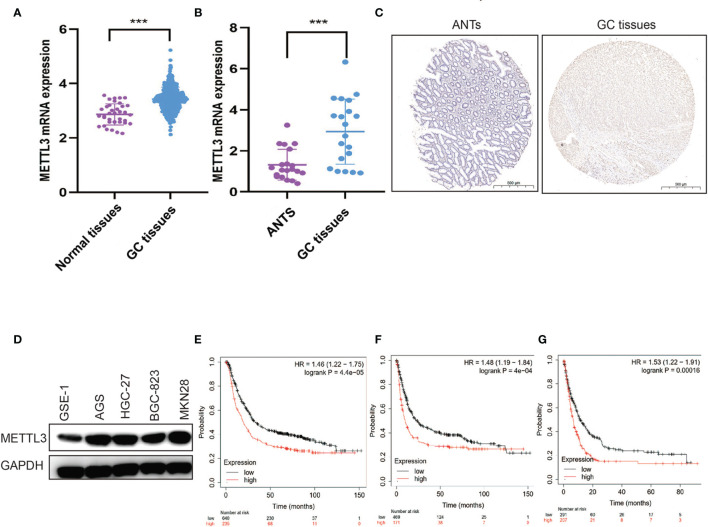
METTL3 is overexpressed and related to poor prognosis in GC. **(A)** METTL3 mRNA expression was evaluated in TCGA GC cohort. **(B)** METTL3 mRNA expression was evaluated in clinical GC samples. **(C)** Representative IHC stains of METTL3 in clinical GC samples. **(D)** METTL3 expression was measured in gastric epithelial cell and GC cell lines. **(E–G)** OS **(E)**, PFS **(F)**, and PPS **(G)** were analyzed in GC patients according to METTL3 expression. ***P < 0.001.

In both clinical and TCGA samples, METTL3 was observed to be highly expressed in tumors (**Figures 1A–C**). Similar results were also detected in different GC cell lines (**Figure 1D**). The bioinformatics analysis revealed that high METTL3 levels were markedly associated with poor PFS, OS, and post-progression survival (PPS) in GC patients (**Figures 1E–G**).

### METTL3 Promotes GC Growth

**Figure 2:**
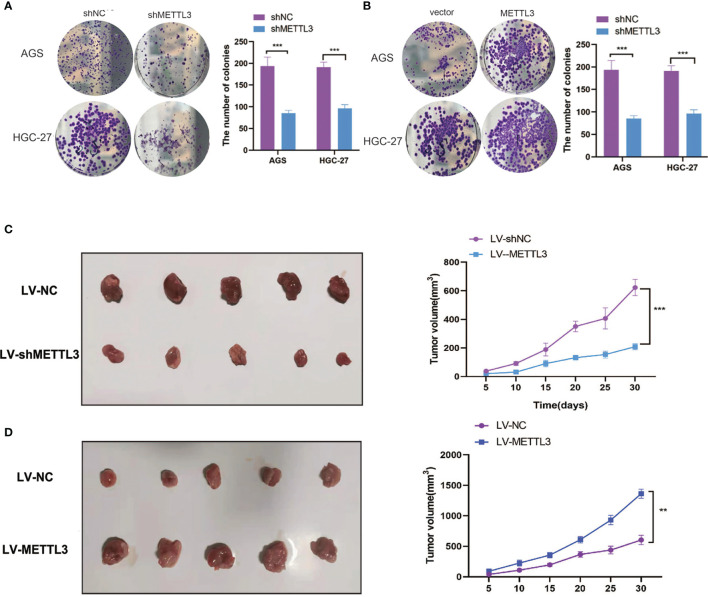
METTL3 promotes GC growth *in vitro* and *in vivo*. **(A, B)** The proliferative ability of GC cells was evaluated after METTL3 knockdown or overexpression by colony formation assay. **(C, D)** The tumor formation was verified after METTL3 knockdown or overexpression in a xenograft model. **P < 0.01, ***P < 0.001.

To confirm the role of METTL3 in human GC, we established stable METTL3 knockdown and overexpression GC cell lines (**Figure S1A**). As expected, METTL3 depletion significantly reduced the m6A levels in GC cells (**Figure S1B**). The CCK-8 and colony formation assays showed that METTL3 overexpression significantly improved GC cell proliferative capacity, while METTL3 deficiency markedly inhibited GC cell survival (**Figures 2A, B** and **Figures S1C, D**). To further confirm our findings *in vitro*, a tumor-bearing mouse model was used to verify the effect of METTL3 on GC. As shown in **Figures 2C, D**, the tumor growth rate was positively correlated with METTL3 expression. Our data confirmed the function of METTL3 in facilitating GC tumorigenicity.

### METTL3 Promotes GC Angiogenesis

**Figure 3:**
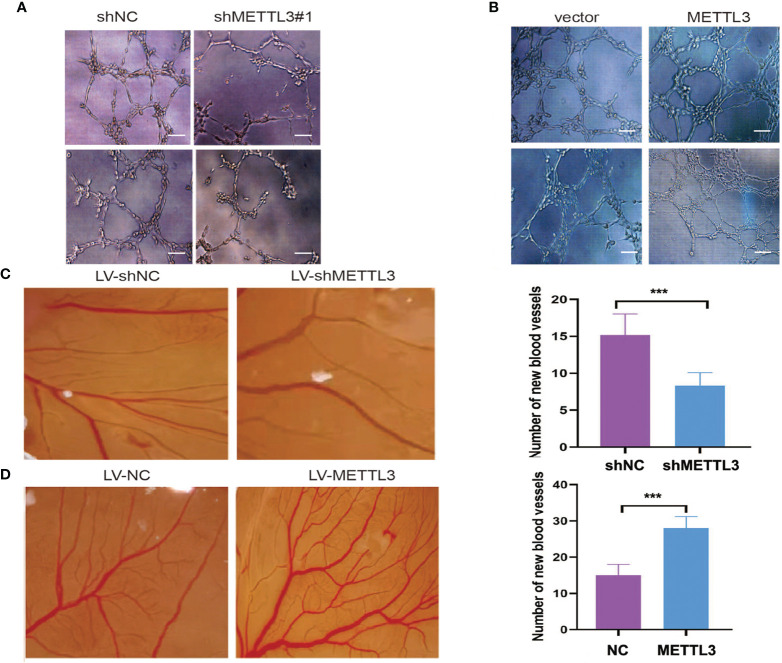
METTL3 promoted GC angiogenesis. **(A, B)** HUVECs cultured with medium obtained from GC cells with METTL3 deficiency or overexpression. **(C)** Representative images of the CAM experiment from LV-shMETTL3 and LV-shNC group. **(D)** Representative images of the CAM experiment from LV-METTL3 and LV-NC group. The average number of new vessels was quantified. Scar bar = 50 μm, ***P < 0.001.

The role of METTL3 in angiogenesis was investigated by using tube formation and CAM assays. As shown in **Figures 3A, B**, HUVEC tube formation was markedly impaired by conditioned medium (CM) from GC cells with METTL3 knockdown, while CM from METTL3-overexpressing GC cells promoted tube formation. In the CAM model, treatment with CM from METTL3 knockdown GC cells obviously decreased the vessel number in comparison with its control group (**Figure 3C**), whereas treatment with CM from METTL3 overexpression GC cells obviously elevated the vessel number in comparison with its control group (**Figure 3D**).

### ADAMTS9 Is Identified as a Downstream Target of METTL3

**Figure 4:**
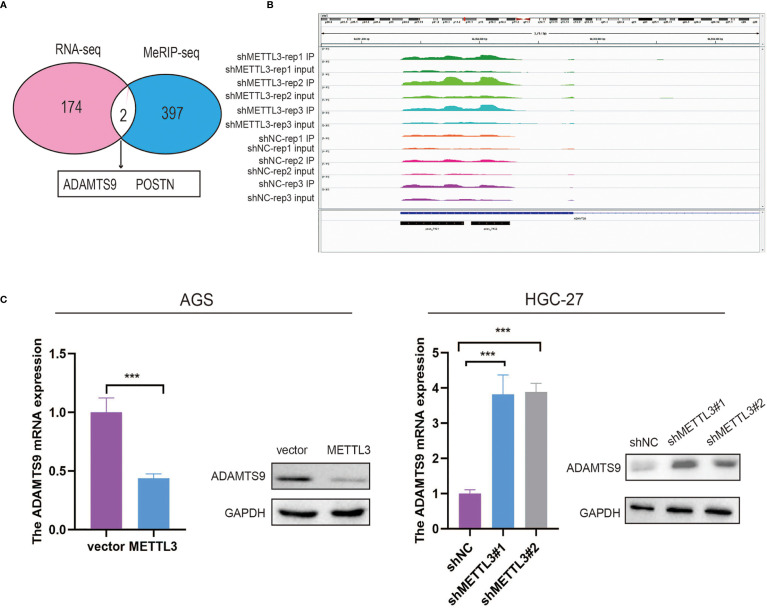
MeRIP-seq and RNA-seq identified ADAMTS9 as a downstream target of METTL3. **(A)** ADAMTS9 and POSTN were selected as candidate genes after filtering the upregulated genes and reduced m6A peaks. **(B)** The m6A abundances on ADAMTS9 mRNA in AGS cells with METTL3 knockdown or not as examined by MeRIP-seq were plotted. **(C)** The expression level of ADAMTS9 in METTL3-decificent and -overexpression GC cells. ***P < 0.001.

In this part, we identified 174 genes that were significantly upregulated in METTL3-deficient GC cells by using RNA-seq (**Figure 4A**). To investigate whether these differentially expressed genes were ascribed to METTL3-mediated m6A modification, MeRIP-seq was employed to map the m6A modification in stable METTL3-knockdown and control GC cells, and 397 peaks were found to be decreased in AGS cells with stable METTL3 knockdown (**Figure 4A** and **Supplementary File 1**). After filtering the upregulated genes and reduced m6A peaks, two genes (ADAMTS9 and POSTN) overlapped between the MeRIP-seq and RNA-seq data (**Figure 4A**). Of the two potential genes, ADAMTS9, which acts as a potential tumor-suppressor gene, was selected for further research (26). Subsequently, an m6A peak was found in ADAMTS9 mRNA in control AGS cells and was decreased upon METTL3 knockdown (**Figure 4B**). Furthermore, we verified that ADAMTS9 was downregulated by METTL3 in GC cells (**Figure 4C**).

### METTL3 Decreased ADAMTS9 mRNA Stability Through an m6A-YTHDF2-Dependent Pathway

**Figure 5:**
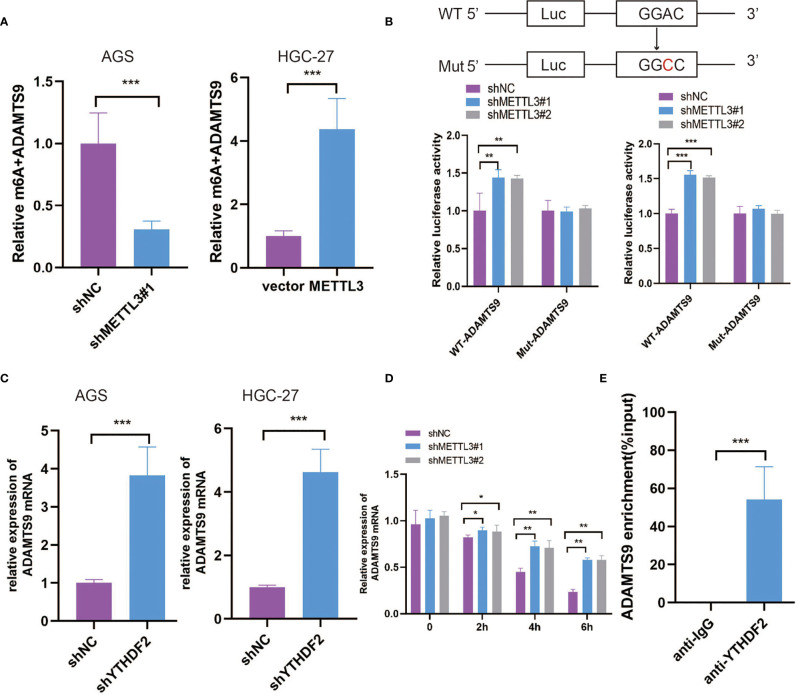
METTL3 decreased ADAMTS9 mRNA stability through m6A-YTHDF2 dependent pathway. **(A)** The regulation of METTL3 on ADAMTS9 m6A modification was evaluated in different GC cells. **(B)** The post-transcription level of ADAMTS9 was detected in METTL3-knockdown GC cells by using firefly luciferase reporter assay. **(C)** The level of ADAMTS9 mRNA was detected in YTHDF2 knockdown GC cells by qRT-PCR. **(D)** ADAMTS9 mRNA was analyzed at indicated times in METTL3 knockdown and control AGS cells with Act-D treatment. **(E)** RIP-qPCR assay was used to detect the enrichment of YTHDF2 binding to ADAMTS9 m6A modification sites. *P < 0.05, **P < 0.01, ***P < 0.001.

MeRIP-seq data were validated to demonstrate the m6A modification of ADAMTS9 mRNA by using MeRIP-qPCR. Our results demonstrated that the m6A-specific antibody markedly enriched ADAMTS9 mRNA in comparison with the IgG control, METTL3 knockdown markedly decreased the m6A levels of ADAMTS9 mRNA in AGS cells, and METTL3 overexpression obviously elevated the m6A levels of ADAMTS9 mRNA in HGC-27 cells (**Figure 5A**). Next, we established luciferase reporters, including the wild-type (WT) and mutant (Mut) reporters. The adenosine (A) bases in the m6A consensus sequence (RRACH) were replaced by cytosine (C) in the mutant form of ADAMTS9. As shown in **Figure 5B**, the luciferase activity of the WT reporter was obviously increased upon METTL3 knockdown, while the Mut reporter exhibited no response to METTL3 knockdown, indicating that the modulation of ADAMTS9 mRNA expression was regulated by METTL3-mediated m6A modification. Previous studies have shown that the m6A-binding proteins YTHDF1/2/3 can target many mRNA transcripts by recognizing m6A motifs (27). In this study, our findings revealed that only knockdown of YTHDF2 could augment ADAMTS9 mRNA expression levels, while YTHDF1/3 had no remarkable effect (**Figure 5C** and **Figure S1E**).

Subsequently, METTL3-knockdown AGS cells were treated with actinomycin D (Act-D) to block RNA transcription. As shown in **Figure 5D**, METTL3 knockdown significantly prolonged the half-life of ADAMTS9 mRNA, which indicated the promoting effect of METTL3 on ADAMTS9 mRNA degradation. YTHDF2-RIP was performed to prove that ADAMTS9 was a target of YTHDF2 (**Figure 5E**), which was reported to target and facilitate the degradation of mRNAs (28). Our data indicated that METTL3 may inhibit ADAMTS9 expression *via* YTHDF2-dependent mRNA decay.

### ADAMTS9 Reversed the Effects of METTL3 in GC

**Figure 6:**
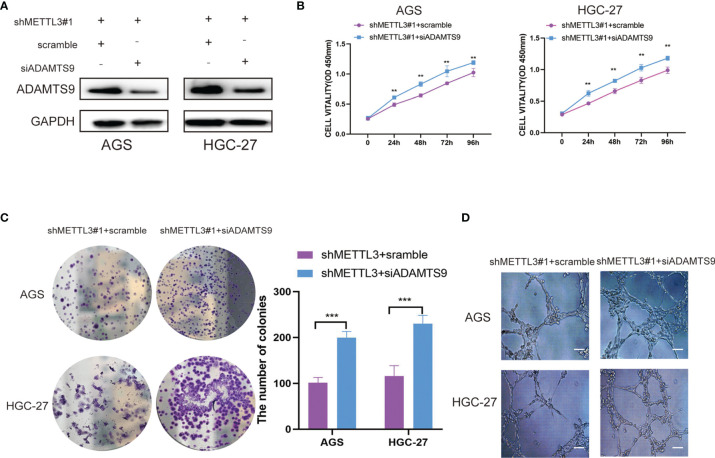
ADAMTS9 reversed the effects of METTL3 in GC. **(A)** The ADAMTS9 expressions were artificially reduced in METTL3 knockdown GC cells. **(B)** The cell viability of METTL3-knockdown GC cells was detected after ADAMTS9 suppression by CCK-8 assay. **(C)** The proliferation of METTL3-knockdown GC cells were detected after ADAMTS9 suppression by colony formation assay. **(D)** The angiogenesis ability of METTL3-knockdown GC cells were evaluated after ADAMTS9 suppression by tube formation assay. Scar bar = 50 μm, **P < 0.01, ***P < 0.001.

To further confirm the importance of ADAMTS9 in our study, we suppressed ADAMTS9 expression in METTL3-knockdown GC cells (**Figure 6A**). The rescue experiments showed that ADAMTS9 inhibition enhanced the proliferative ability of METTL3-knockdown GC cells (**Figures 6B, C**). In addition, ADAMTS9 suppression also reversed the inhibition of HUVEC tube formation affected by METTL3 deficiency (**Figure 6D**). Therefore, our work demonstrated that METTL3 facilitates angiogenesis and carcinogenesis in GC by reducing the expression of ADAMTS9.

### METTL3 Promotes GC Progression Through ADAMTS9-Mediated PI3K/AKT Signaling

**Figure 7:**
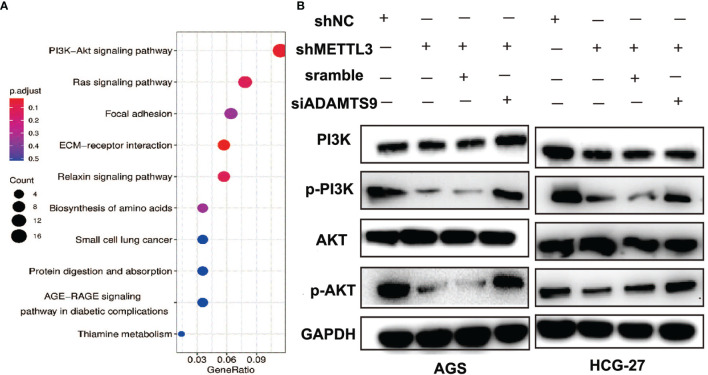
METTL3 promotes GC progression through ADAMTS9-mediated PI3K/AKT signaling. **(A)** Top 10 enrichment KEGG pathways of dysregulated genes in METTL3-knockdown GC cells. **(B)** The activation of PI3K/AKT signal pathway was evaluated using Western blot in GC cells with indicated gene knockdown. GAPDH was used as a loading control.

Through RNA-seq analysis, we found that the downregulation of METTL3 could cause a large number of changes in gene expression levels, and the top 10 KEGG pathways of differentially expressed genes caused by METTL3 knockdown are shown in **Figure 7A**. Among them, the PI3K/AKT signaling pathway was included. A previous study showed that ADAMTS9 exerted antitumor effects by inhibiting the PI3K/AKT/mTOR pathway in GC (26). In our work, the PI3K/AKT signaling pathway was significantly inactivated due to the downregulation of PI3K and AKT phosphorylation in METTL3-knockdown GC cells. Moreover, ADAMTS9 inhibition reactivated the phosphorylation of PI3K and AKT (**Figure 7B**). These results indicated that METTL3 might facilitate GC progression through ADAMTS9-mediated PI3K/AKT signaling.

## Discussion

The clinical relevance and functional role of METTL3 in malignancies are controversial. In most studies, METTL3 has been recognized as a potential oncogene in human cancers (29). However, few studies have also provided the converse findings regarding METTL3 expression and its tumor-suppressing role in endometrial and breast cancer (30, 31). A recent study indicated that METTL3 is overexpressed in GC patients who have a shortened survival time (32). Another study demonstrated that the METTL3/BATF2/p53/ERK axis suppresses GC development by modulating cell-cycle progression and extracellular matrix degradation (33). Consistently, our findings support METTL3 as a candidate oncogene that is markedly upregulated and dramatically associated with poor outcome in GC.

Previous studies have found a series of molecular targets of METTL3 involved in the carcinogenesis of GC, such as HDGF (34) and SEC62 (35). In a recent study, the METTL3/SPHK2/KLF2 axis was demonstrated to promote the proliferation and metastasis of GC cells (36). Another study revealed that THAP7-AS1, modified by METTL3-mediated m6A, plays an oncogenic role in GC cells by repressing the transcription of miR-320a and miR-22-3p (37). In the present study, our data suggested that ADAMTS9 could be a new potential downstream target of METTL3 involved in GC carcinogenesis and angiogenesis. ADAMTS9 belongs to the ADAMTS protein family and is frequently downregulated as a result of promoter hypermethylation in a variety of human cancers (38). In addition, ADAMTS9 is considered to be an independent prognostic factor of GC (26). Our findings might be conducive to gaining a better understanding of the mechanisms of METTL3 in GC pathogenesis.

YTHDF1/2/3 are the core reading proteins for m6A modification. The relationship between ADAMTS9 and YTHDF family proteins is unclear. In a recent study, ADAMTS9 was found to regulate mRNA decay *via* the m6A/mRNA pathway as a target of YTHDF2 in spermatogenesis (39). Here, we consistently confirmed the regulatory role of YTHDF2, but not YTHDF1/3, on ADAMTS9 in GC. Briefly, YTHDF2 modulated the ADAMTS9 mRNA half-life in a METTL3-dependent manner, which may interfere with the antitumor functions of ADAMTS9. Our work provides novel insights into METTL3-mediated GC growth and angiogenesis *via* the YTHDF2/ADAMTS9 axis.

In addition, ADAMTS9 has been identified to inhibit tumor progression by regulating the PI3K/AKT/mTOR pathway (40, 41). The latest studies have shown that PI3K/AKT/mTOR can also be activated by METTL3-mediated M6A modification in ovarian cancer (42) and retinoblastoma (43). Here, by analyzing differentially expressed genes caused by METTL3 knockdown, the PI3K/AKT signaling pathway was proven to be correlated with METTL3 expression in GC. Further experiments verified that p-PI3K and p-AKT were downregulated after METTL3 knockdown, while ADAMTS9 inhibition increased the expression of p-PI3K and p-AKT in GC cells. Our results indicated that METTL3 might facilitate GC progression through the ADAMTS9-mediated PI3K/AKT signaling pathway.

In conclusion, we demonstrated a new METTL3-related signaling pathway that facilitates angiogenesis and carcinogenesis in GC. In brief, METTL3 overexpression in GC promoted the m6A modification of ADAMTS9, a tumor-suppressor gene, and epigenetically prevented its transcription in a YTHDF2-dependent manner. This work sheds light on the pathological role and molecular mechanism of METTL3 and further supports that METTL3 could be a candidate prognostic biomarker and therapeutic target for GC.

## Data Availability Statement

The datasets presented in this study can be found in online repositories. The names of the repository/repositories and accession number(s) can be found in the article/**Supplementary Material**.

## Ethics Statement

The studies involving human participants were reviewed and approved by the ethics committee of Nanjing First Hospital. The patients/participants provided their written informed consent to participate in this study. The animal study was reviewed and approved by the ethics committee of Nanjing First Hospital.

## Author Contributions

XW, HX, and XS designed the experiments. NW, XH, and XC performed the experiments. NW, SZ, and XH analyzed and confirmed all data and wrote the manuscript. BZ assessed all content. All authors reviewed the manuscript. XW and HX made a final approval. All authors contributed to the article and approved the submitted version.

## Funding

This work was supported by Grants from the National Natural Science Foundation of China (Grant No. 81773240), The Natural Science Foundation of Jiangsu Province (Grant No. BK20181118), Jiangsu Provincial Special Program of Medical Science (BE2019617), and Nanjing Medical Science and Technique Development Foundation (Grant No. QRX17062).

## Conflict of Interest

The authors declare that the research was conducted in the absence of any commercial or financial relationships that could be construed as a potential conflict of interest.

The reviewer YY declared a shared parent affiliation with the authors to the handling editor at the time of review.

## Publisher’s Note

All claims expressed in this article are solely those of the authors and do not necessarily represent those of their affiliated organizations, or those of the publisher, the editors and the reviewers. Any product that may be evaluated in this article, or claim that may be made by its manufacturer, is not guaranteed or endorsed by the publisher.

## Supplementary Material

Supplementary Figure 1 **(A)** Efficient knockdown and overexpression of METTL3 were established in GC cells. **(B)** The m6A content of total RNAs in METTL3 knockdown GC cells. **(C)** The proliferative ability of GC cells after METTL3 knockdown. **(D)** The proliferative ability of GC cells after METTL3 overexpression. **(E)** The ADAMTS9 mRNA expression levels were evaluated in GC cells after YTHDF1 or 3 knockdown.
