# Supplementary figures and images for: METTL3-Mediated ADAMTS9 Suppression Facilitates Angiogenesis and Carcinogenesis in Gastric Cancer
Source: Front Oncol. 2022 Apr 28;12:861807. doi: 10.3389/fonc.2022.861807 (PMC9097454; doi:10.3389/fonc.2022.861807)

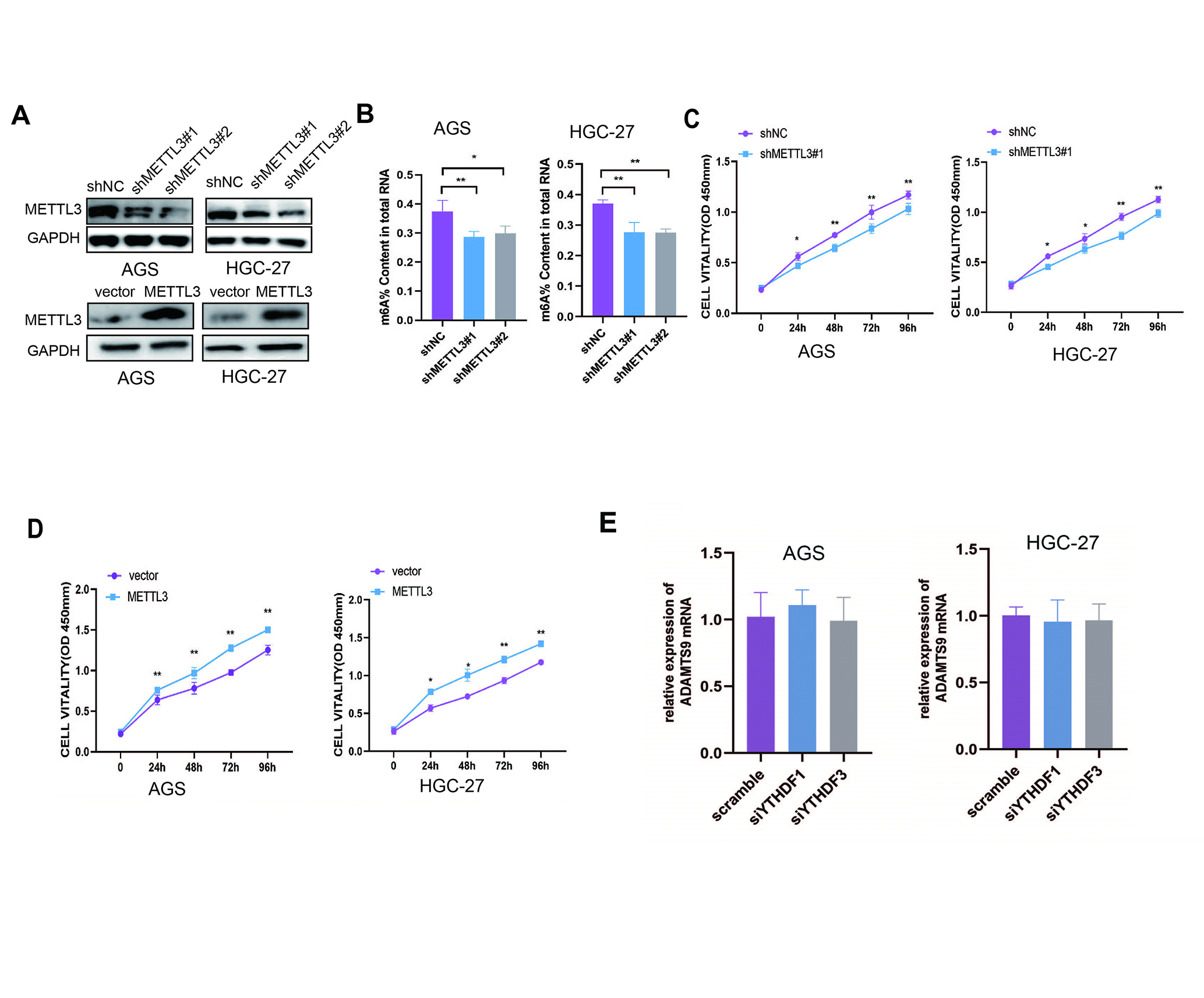

Supplement: Supplementary Figure 1 — (A) Efficient knockdown and overexpression of METTL3 were established in GC cells. (B) The m6A content of total RNAs in METTL3 knockdown GC cells. (C) The proliferative ability of GC cells after METTL3 knockdown. (D) The proliferative ability of GC cells after METTL3 overexpression. (E) The ADAMTS9 mRNA expression levels were evaluated in GC cells after YTHDF1 or 3 knockdown. [file Image_1.tif]
